# Putative Monofunctional Type I Polyketide Synthase Units: A Dinoflagellate-Specific Feature?

**DOI:** 10.1371/journal.pone.0048624

**Published:** 2012-11-05

**Authors:** Karsten Eichholz, Bánk Beszteri, Uwe John

**Affiliations:** Alfred Wegener Institute for Polar and Marine Research, Bremerhaven, Germany; Duke University Medical Center, Duke University, United States of America

## Abstract

Marine dinoflagellates (alveolata) are microalgae of which some cause harmful algal blooms and produce a broad variety of most likely polyketide synthesis derived phycotoxins. Recently, novel polyketide synthesase (PKS) transcripts have been described from the Florida red tide dinoflagellate *Karenia brevis* (gymnodiniales) which are evolutionarily related to Type I PKS but were apparently expressed as monofunctional proteins, a feature typical of Type II PKS. Here, we investigated expression units of PKS I-like sequences in *Alexandrium ostenfeldii* (gonyaulacales) and *Heterocapsa triquetra* (peridiniales) at the transcript and protein level. The five full length transcripts we obtained were all characterized by polyadenylation, a 3′ UTR and the dinoflagellate specific spliced leader sequence at the 5′end. Each of the five transcripts encoded a single ketoacylsynthase (KS) domain showing high similarity to *K. brevis* KS sequences. The monofunctional structure was also confirmed using dinoflagellate specific KS antibodies in Western Blots. In a maximum likelihood phylogenetic analysis of KS domains from diverse PKSs, dinoflagellate KSs formed a clade placed well within the protist Type I PKS clade between apicomplexa, haptophytes and chlorophytes. These findings indicate that the atypical PKS I structure, i.e., expression as putative monofunctional units, might be a dinoflagellate specific feature. In addition, the sequenced transcripts harbored a previously unknown, apparently dinoflagellate specific conserved N-terminal domain. We discuss the implications of this novel region with regard to the putative monofunctional organization of Type I PKS in dinoflagellates.

## Introduction

Dinoflagellates are among the most important primary producers and therefore are important drivers of marine food webs. They also play a crucial role in structuring microbial plankton communities due to their auto- and heterotrophic or even parasitic life styles. Apart from that, some of them are the major causative agents for red tides and harmful algal blooms (HAB) in marine environments. Dinoflagellates produce a wide variety of most likely polyketide synthesis derived secondary metabolites among which several well described phycotoxins can elicit life-threating symptoms in humans [1]–[3].

Polyketides are a structurally diverse class of secondary metabolites with various potential biomedical or pharmaceutical applications as antibiotics, insecticides, immunosurpressive and anti-tumor agents [4]. The diverse and complex structures of these compounds are assembled through repetitive condensation and reduction steps of simple acyl monomers through polyketide synthases (PKS) [4]. PKS are large multi-domain enzyme complexes which resemble fatty synthases (FAS) both in structure and function and most probably also share a common origin with the latter [5]. In general, PKS require a minimum set of catalytic domains which are ketoacylsynthase (KS), acyl transferase (AT) and acyl carrier protein (ACP) for one round of chain extension. Beyond that, the presence of ketoacylreductase (KR), dehydrases (DH) and enoylreductases (ER) may lead to a stepwise reduction of keto-groups and thus contribute to the vast variety of polyketide structure [4].

PKS are traditionally classified into three types. Type I PKS are large multifunctional proteins which combine several domains in one protein. Two subclasses are known for Type I PKS. Fungal iterative Type I PKSs use the same set of catalytic domains on one protein several times for chain extension, analogously to vertebrate FAS. In contrast, modular Type I PKS function in a conveyor belt like manner in that the different catalytic domains are organized in modules comprising all required enzymatic functions. Each module is used only once during polyketide assembly, thus the architecture of each module influences the overall polyketide structure. For Type II PKSs, the catalytic domains are spread on iteratively used monofunctional proteins which form complexes for the assembly of polyketides. The Type III PKS form monofunctional, homodimeric proteins and do not require ACP but act directly on acetyl-Coenzyme A during synthesis [6].

Type I PKS genes have been identified in both prokaryotes and eukaryotes. They have been reported from fungi and bacteria [4] where their phylogenetic relationships reflect both their source organisms and functional organization [5]. In the last decade, Type I PKS have also been found in the genomes of various unicellular eukaryotes such as apicomplexa (alveolates) [7], haptophytes (stamelopiles) [8]–[10] and chlorophytes (plantae) [10] as well as in a dinoflagellate species (alveolates) [11]. Interestingly, an evolutionary interrogation of Type I PKS from apicomplexa, haptophytes and chlorophytes revealed that they form their own distinct protistan PKS clade within the PKS/FAS evolution [10]. Nevertheless, the phylogenetic relationships within this clade do not mirror the proposed evolution of their source species [10], [12].

In 2008, eight full length PKS transcripts showing phylogenetic affinities to apicomplexan modular Type I PKS were sequenced from the toxic dinoflagellate *Karenia brevis* [11]. Unexpectedly, these transcripts showed what appeared to be a novel architecture for Type I PKS in that 7 out of 8 PKS related transcripts carried only a single KS domain [11], [13]. This monofunctional organization has generally been seen as a feature typical of Type II PKS [4]. On the other hand, transcripts encoding at least two domains have also been observed, both in *K. brevis* (ACP-KS) [11] and later in *Heterocapsa circularisquama* (KS-KR) [14]. Thus, although the presence of typical PKS I structure (expression as large multi-domain complexes) in dinoflagellates is still conceivable, these protists definitely seem to also harbor PKS I like genes apparently expressed as monofunctional units which does not correspond to the previous understanding of Type I polyketide synthases [11], [13]. So far, full length Type I PKS mRNA data have only been obtained from gymnodiniales *K. brevis*, and an evolutionary classification of a comprehensive dinoflagellate KS data set encompassing representatives from different dinoflagellate orders has not been done.

Here, we aimed to gain further insights into the organization of PKS genes in dinoflagellates and to resolve the phylogenetic relationships of dinoflagellate PKS with respect to other available protistan representatives. By sequencing full length transcripts from two further species representing other major groups within dinoflagellates, *Alexandrium ostenfeldii* (gonyaulacales) and *Heterocapsa triqueta* (peridiniales), we address the question concerning the number of PKS related domains per expression unit in dinoflagellates. With respect to PKS evolution, we performed maximum likelihood analysis in order to test the hypothesis that dinoflagellate PKS derive from Type I PKS and form their own phylogenetic clade indicating a common evolutionary origin. Our findings support the hypothesis that the monofunctional Type I PKS organization is widespread in dinoflagellates.

## Results

### PKS Sequences in Dinoflagellates

The dinoflagellate SL has been shown to denote the 5′ end of dinoflagellate mRNA in several publications and has been used here as a target sequence in 5′ RACE PCR [15]–[18]. Using a *K. brevis* KS domain sequence as a query, we identified five similar sequences in publicly available EST data from *A. ostenfeldii* [18] and *H. triquetra* [19] Rapid amplification of cDNA ends was done using gene specific primers designed for these sequences. This resulted in five full length mRNA sequences with an average length of 2988±157 bases (mean ± std. error, n = 5) (three from *A. ostenfeldii* KS (accession numbers: Ac0019: JX068836, Ac0038: JX068837, 10-x_J14: JX068838) and two *H. triquetra* KS (Accessionnumber: HTE5908: JX068839, HTE6310:JX068840) (Table 1). All transcripts were characterized by the presence of the dinoflagellate specific spliced leader sequence, the eukaryote specific polyA tail and 3′UTRs of 120±64 bases (mean ± std. error, n = 5) length in average. BLASTx analysis of the full length transcripts yielded high similarities to *K. brevis* KS KB2006 in four cases and high similarity to *K. brevis* KS KB 5361 in one case (Table 1).

**Table 1:** Sequence properties of the ketoacyl snthases transcripts identified in *A. ostenfeldii* and *H. triquetra.*

Sequence ID	Length (Base Pairs)	orf	Amino Acids	best BlastX hit	Identity/Similarity (%)/(%)	Accession Number
Ac0019	3018	106–2931	935	KB2006	35/51	ABQ85797
Ac0038	3148	87–2915	938	KB2006	59/74	ABQ85797
10-x_J14	2729	127–2619	821	KB5361	32/49	ABQ85800
HTE5908	3059	100–2958	969	KB2006	30/48	ABQ85797
HTE6310	2988	86–2917	943	KB2006	57/73	ABQ85797

**Figure 1:**
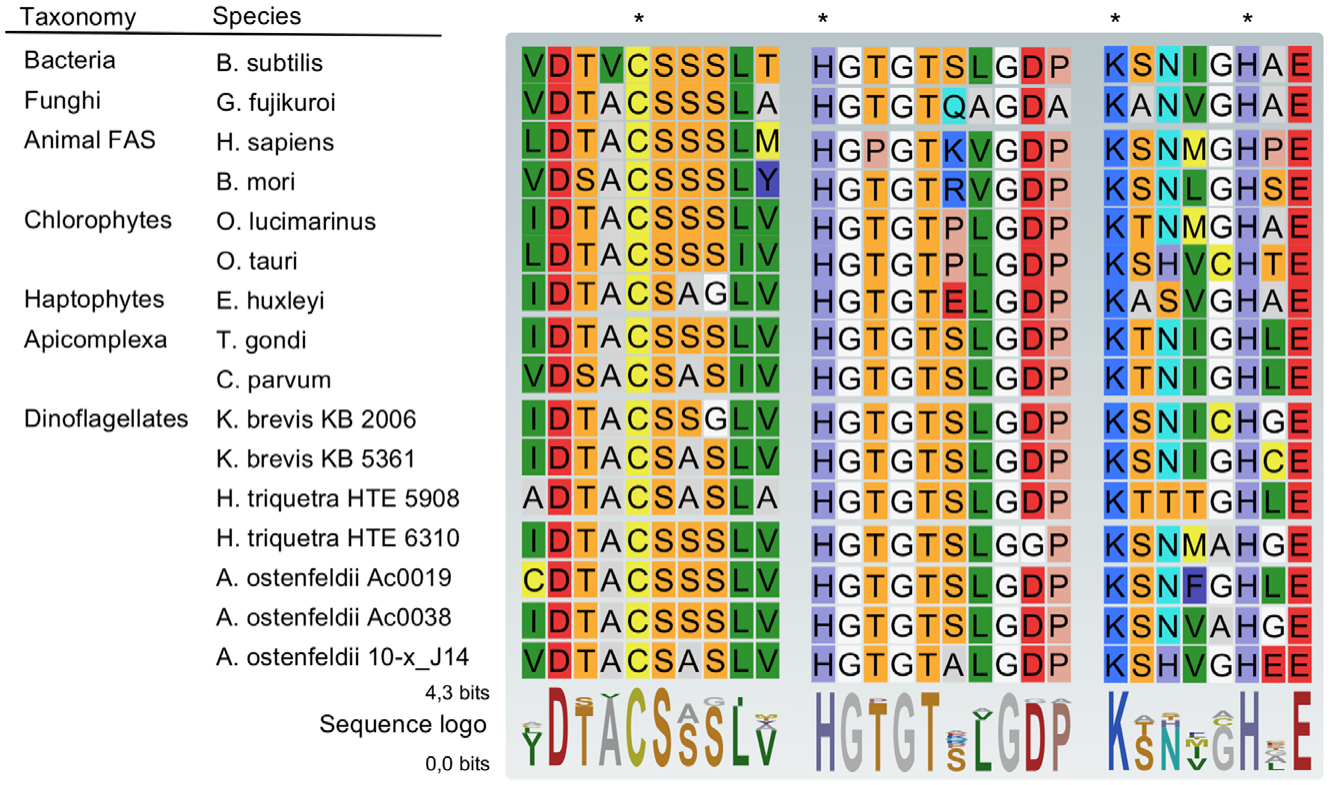
Conserved active site motifs of the ketoacyl synthase domain from different evolutionary distinct supergroups. A special emphasis was thereby given to protistan Type I PKS (Chlorophyta, Haptophyta, Apicomplexa and Dinoflagellata). A sequence logo of the active site motifs is given beneath the alignment; the height of the letters thereby indicates the degree of conservation. The three *A. ostenfeldii* KS sequences (Aost ac0019, Aost ac0038, and Aost 10-x_J14) and the two *H. triquetra* KS sequences (Hcaps HTE 5908 and Hcaps HTE 6310) contain all four active site amino acids [20] as is indicated by an asterisk.

*In silico* translation of the transcripts resulted in an average protein size of approximately 100 kDa. The presence of a KS domain was confirmed for all five candidates by a HMMER comparison with PFAM profiles. Notably, none of the transcripts contained more than one PKS related domain. We verified that the characteristic functionally important amino acid (aa) residues are present in the active sites [20]. The important cysteine was present within the DTACSS-motif, as well as both histidine and lysine residues required for catalysis in all five KS sequences (Fig. 1).

### Dinoflagellate PKS are Indeed Expressed in Discrete Domains

To further support the hypothesis of a global monofunctional organization of PKS in dinoflagellates, Western Blots were performed using a dinoflagellate KS specific antibody targeting KS in protein extracts from *A. ostenfeldii* and *H. triquetra* showing a distinct band for both species at predicted size (approximately 100 kDa) for a single KS protein (Fig. S1).

### Dinoflagellate KS Form a Clade within PKS Evolution

**Figure 2:**
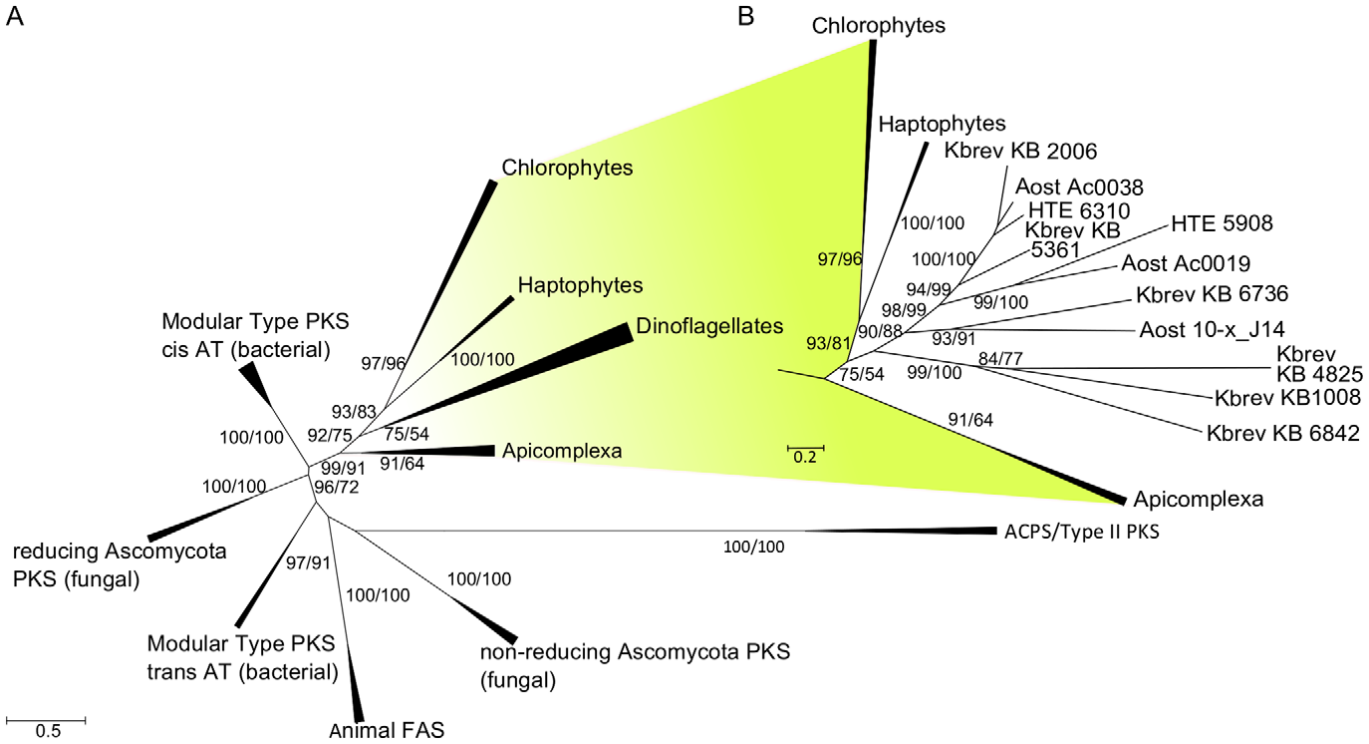
Phylogenetic tree of Type I and Type II KS domains from prokaryotic and eukaryotic PKS and FAS. Fifty-nine taxa representing Type I and Type II KS domains were analyzed by a maximum likelihood approach. The Type II KS and the acyl carrier protein synthases (ACPS) were used as outgroup. Approximate likelihood ratios ≥50% and bootstrap values ≥50% are marked on appropriate branches like (50/50). A. Overall tree topology is in accord with previous reports [5], [10]. Dinoflagellate KS is classified as a well supported group within the protistan Type I FAS/PKS clade. B. Within the dinoflagellate KS group, one clade containing sequences from different species and one only from *K. brevis* can be observed.

Maximum likelihood phylogenetic analysis of the obtained KS sequences placed them well among other Type I PKS. An evolutionary relationship of *K. brevis* PKS to apicomplexan modular Type I PKS has been shown previously [11] and protistan Type I PKS form distinct monophyletic clades within PKS/FAS evolution [10]. Our phylogeny resolved the previously known basic topology (Fig. 2A) [5], [10]. Type II PKS and β-ketoacyl acyl carrier protein synthases (ACPS) were chosen as outgroup and formed a clade with 100% approximate likelihood ratio (aLRT) and bootstrap support (BT). All dinoflagellate KS fragments analyzed grouped well within the strongly supported PKS I clade, underlying that in spite of the organizational structure resembling PKS II, these fragments are evolutionarily correctly classified as PKS I. The phylogenetic tree shows a strongly supported protistan clade (99% aLRT/91% BT) which consists of apicomplexa, dinoflagellates, haptophytes as well as chlorophytes and is clearly separated from the other major groups in Type I FAS/PKS evolution. Within the protistan group, distinct well supported clades are formed. The apicomplexa diverged first in this evolutionary model (91% aLRT/64% BT), followed by the dinoflagellates which have moderately to low support (75% aLRT/54% BT). Within the dinoflagellate clade (Fig. 2B), two well supported groups can be observed (99% aLRT/100% BT and 90% aLRT/88% BT). One group contains only KS sequences from *K. brevis* while the other one is mixed and compromises KS sequences from *A. ostenfeldii*, *H. triquetra* and *K. brevis*.

### Putative Dinoflagellate KS Contain Features of Unknown Function

**Figure 3:**
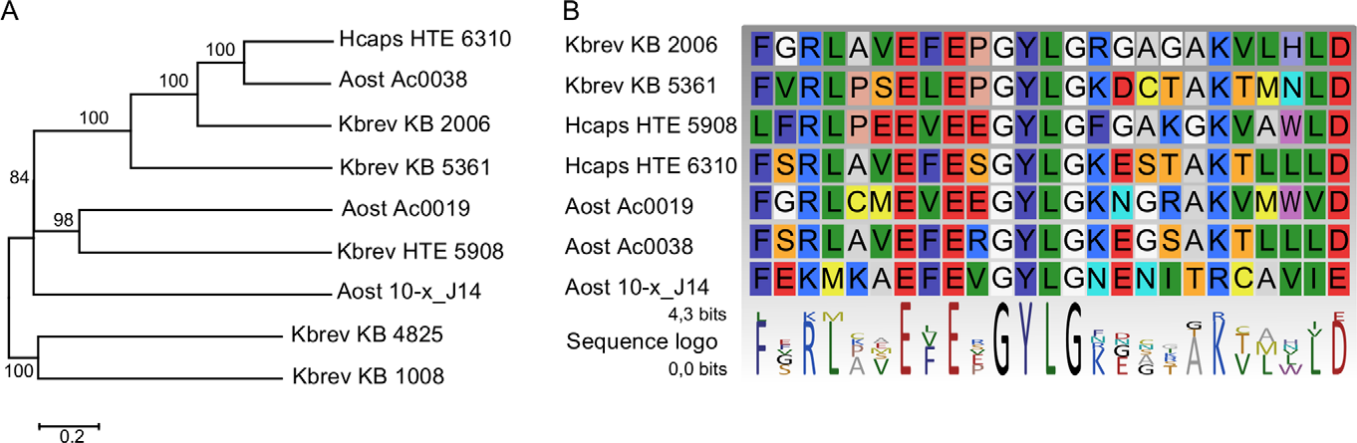
Phylogena and multiple alignment of the truncated N-terminal region from dinoflagellate KS. A. Maximum likelihood phylogenetic analysis of truncated N-terminal regions with 100 Bootstrap repetions. B. Multiple alignment of a highly conserved motif within the N-terminal region and the sequence logo showing the degree of sequence conservation of the respective site.

The KS domains previously described from K. brevis (KB2006 and KB 5361) as well as those 5 sequences described here have a mean size of 389±21 aa (mean ± std. error, n = 7) and are located in the center of the respective protein sequences. The complete length of these putative proteins was estimated as 915±54 (aa mean ± std. error, n = 7). The central KS domain is in each case flanked by a longer N-terminal (340±15 aa mean ± std. error, n = 7) and a shorter C-terminal sequence segment (185±67 aa mean ± std. error, n = 7). Neither BLAST nor PFAM domain search revealed any significant similarity to previously characterized proteins/domains. Nevertheless, these amino acid sequences showed similarities to the respective region of K. brevis KS sequences from public databases. The sequence identities for the A. ostenfeldii and H. triquetra N-terminal regions to K. brevis KB2006 ranged from 25% to 53%. A maximum likelihood phylogeny (Fig. 3A) of the truncated N-terminal region showed a close relationship of the A. ostenfeldii and H. triquetra N-terminal regions to the corresponding regions from Karenia brevis KB 2006 and KB 5361. Like in the maximum likelihood phylogenetic analysis of the KS domain (Fig. 2A and 2B), we found a well supported mixed clade containing sequences from all three species and one well supported clade containing sequences from K. brevis only. Both topologies are almost identical, albeit we did not include the K. brevis sequences KB 6842 and KB 6736 in our analysis of the N-terminal region because in these transcripts, the KS domain is directly located at the N-terminus. Throughout the alignment of this N-terminal region, numerous highly conserved positions can be observed. In particular, a central conserved ExExGCLG motif caught our interest which was neighbored by several more conserved amino acids (Fig. 3B). We also analyzed the C- terminal regions in a similar manner, calculating a multiple sequence alignment and a phylogeny. In this region, we could not detect any conserved patterns in the multiple alignment. The tree topology was well supported but differed substantially in its structure (Data not shown) from the trees of the KS domain and the N-terminal region.

## Discussion

### Origin and Functional Organization

We sequenced and analysed five novel full length KS transcripts from *A. ostenfeldii* and *H. triquetra*. An origin from bacterial contamination of these KS candidates can be excluded as the obtained sequences all include 3′-UTRs and poly(A^+^) tails as typical eukaryotic feature. The presence of the DinoSL at the 5′end further substantiates their dinoflagellate origin and the integrity of the 5′end [21]. The dinoflagellate origin was also confirmed by phylogenetic analysis in which the novel KS sequences grouped together with the previously described KS sequences from *K. brevis* within a protistan clade [11]. In a recent study, partial sequences of hybrids of non-ribosomal peptide synthetases and PKS genes have been found in *K. brevis* [22]. Differently from the PKS genes found by Monroe & van Dolah (2008) and this study, those genes obviously originated via gene transfer from cyanobacteria [22]. We could also confirm the presence of cyanobacterial related PKS clades in dinoflagellates by implementing these sequences into our evolutionary model (Fig. S2), but did not include this sequences into our final analysis due to sequence length inconsistencies.

This is the second report of full length PKS transcripts from dinoflagellates. Previously, full length PKS transcripts have only been identified and characterized in *K. brevis* [11], [13]. All five KS sequences contain the key motifs and amino acids in the respective site required for catalysis [20] and are therefore likely involved in condensation processes accomplished by PKS. Based on our evolutionary analysis and the sequence annotation but in the absence of any functional assay to prove the condensation capacity of these putative enzymes, we refer to them as putative monofunctional Type I PKS domains. Furthermore, we cannot link the different KS genes to the production of specific polyketides (e.g. phycotoxins like brevetoxin or spirolides) due to the apparent monofunctional organization, the absence of functional assays and patchy species distribution within the dinoflagellate KS clade (Fig. 2B).

We demonstrated both at the transcript and protein level that these putative PKS constituents are expressed as monofunctional units. This feature seems to be unique to dinoflagellates. Even other chromalveolates, like *Cryptosporidium parvum* (Apicomplexa) or *Emiliana huxleyi* (haptophytes), display classic modular Type I PKS proteins with several different enzymatic domains organized into distinct modules within what appears to be a single transcriptional and translational unit [7], [10]. However, there are also reports on partial PKS transcripts in dinoflagellates containing more than one PKS domain, although the typical, complete modular structure has not been reported from them as yet [11], [14]. Thus, the emergence of PKS I expressed as monofunctional unit can presumably be dated around the origin of the dinoflagellate group.

This poses the question why and how the most probably multifunctional PKS proteins have evolved into the observed monofunctional organization in dinoflagellates. The complexity, the enormous size of the dinoflagellate genome and the low gene density might play a role in this context [18], [23]. The dinoflagellate genome is interspersed with a large number of simple and complex repeats [18], [23] which are well known for frequent recombination events. Furthermore, it is a widespread phenomenon that dinoflagellate genomes harbor genes in high copy numbers (up to ∼2000 in some cases) [24] indicating a considerable number of gene duplication events in dinoflagellate evolution. In the course of evolution, this might have led to the decomposition of the Type I PKS gene clusters as these have also been shown to undergo frequent shuffling of their domains and modules [5]. Earlier reports from bacterial modular Type I PKS already showed that this multifunctional structure can at least partially disaggregate [6]. Beyond that, a SL mediated retroposition of mRNA back into the genome has been postulated for dinoflagellates, a mechanism enabling frequent shuttling of reverse transcribed mRNA back into the genome [17], [18]. Such a mechanism could also favor shorter inserts thus leading to a decline of the modular PKS model towards a monofunctional organization. Evidentially, our findings show that the traditional classification of PKS into I, II and III based on their functional organization is not fully congruent with a phylogenetic classification [4], [6].

### Evolutionary Classification

The addition of our novel sequences from *A. ostenfeldii* and *H. triquetra* to the KS phylogenetic dataset provides strong evidence for a unique dinoflagellate KS clade within PKS gene evolution. Also other phylogenetic groups (haptophytes, chlorophytes and apicomplexa) that diverged early on in eukaryote evolution retained evolutionary signal in their KS domains and remained in well supported monophyletic classes within the tree. This is consistent with earlier findings regarding the evolution of protistian PKS [10] and the evolutionary affinity of *K. brevis* KS and Apicomplexan PKS [11]. In dinoflagellates, we observed two clades. One comprised sequences from three dinoflagellates with KS typical amino acid motifs, whereas the other contained only KS sequences from *K. brevis* which show alterations in their active sites [11]. This topology indicates a history with early gene duplications within the dinoflagellate clade. Nevertheless, improved sampling of both taxa and their KS types will be needed before this history can be resolved with any substantial confidence.

### Flanking Regions

The dinoflagellate KS proteins identified so far exhibit less conserved flanking regions on usually both sides of the conserved KS domain. In the N-terminal region, at least one highly conserved amino acid motif was identified. The function of this motif cannot be determined based on homology, as it shows no significant similarity to any other currently available sequences and therefore, this signature N-terminal motif seems to be unique to dinoflagellates thus far. The C-terminal region does not show conserved aa structure in the alignment and harbors rather different phylogenetic signal from both the KS domain and the N-terminal flanking region. One could speculate that the function of the N-terminal region might be related to the monofunctional organization of these KS. We propose three possible functions. First, the N-terminal region might be required for structural purposes. If the PKS in dinoflagellates derives from modular Type I PKS, the loss of adjacent protein domains might have led to structural rearrangements of the 3D protein structure affecting the enzymatic activity. In this case, the N-terminal region could be a structural replacement for absent adjacent PKS domains and the conserved motifs might be crucial for enabling a correct folding of the polypeptides. Second, the N-terminal region could have a substrate docking function in either delivering the growing polyketide or fatty acid to the KS domain like an ACP or AT. And third, the N-terminal region might be a protein-protein interaction site in which case the motifs could be required to recognize binding partners during the assembly of the different enzymes involved in assembly of a polyketide.

Analysis of N- and C- terminal docking domains of modular Type I PKS and non-ribosomal polypeptide synthases has shown that such linker structures do exist for multifunctional enzymes. However, the linker regions observed there are much shorter (below 100 amino acids) than our N- and C- terminal regions [25]. In 6-deoxyerythronolide B synthase, a modular Type I PKS in *Saccharopolyspora erythraea,* the N-terminal docking domains encompass approximately 120 amino acids in length upstream of the first KS domain [26]. The N-terminal region of dinoflagellate KS is substantially longer (389±21 aa mean ± std. error, n = 7) and shows a high degree of sequence conservation over its complete length. This is generally not the case for the linker sequences, therefore an enzymatic or structural function for these regions are more plausible assumptions. For the C-terminal region, the function cannot be assigned, however, due to size variation and diverse aa sequence composition it might play a role as a linker.

### Conclusions

We characterized five novel full length transcripts encoding putative monofunctional PKS KS proteins from the dinoflagellates *A. ostenfeldii* and *H. triquetra*. We provide evidence that 1) they are evolutionarily correctly classified as Type I PKS; 2) untypical for this class of PKS, but similarly to the situation previously reported from *K. brevis*, they are expressed as monofunctional units. Thus, Type I PKS expressed as monofunctional units seems to be a feature widespread in dinoflagellates, providing one more example of the limitations of the traditional type I, II and III paradigm for classifying PKS [6]. We also identified a previously uncharacterized conserved motif with unknown function in the N-terminal region of the predicted proteins. It can be speculated that the function of these motifs might be related to the unusual functional organization of these dinoflagellate PKS units.

## Materials and Methods

### Dinoflagellate Cell Culture, Nucleic Acid and Protein Extraction

The *Alexandrium ostenfeldii* (AOSH 2, originally isolated from North Atlantic, kindly provided by Nancy Lewis, National Research Council, Halifax, Canada) and *Heterocapsa triquetra* (SCCAP strain K-0481) strains were cultured at 15°C under cool-white fluorescent light at a photon flux density of 40 µmol photons m^−2^ s^−1^ and a 16 h:8 h light:dark photocycle. The dinoflagellate cultures were grown in 500 mL Erlenmeyer flasks in K-medium [27] prepared from sterile-filtered (VacuCap 0.2 µm, Pall Life Sciences) natural North Sea seawater (salinity 32 PSU).

Total RNA and protein fraction was isolated from dinoflagellate cultures using TriReagent® (Invitrogen, Darmstadt, Germany) according to the manufacture’s protocol. Algal cells were harvested by separation over 10 µm gauze to reduce bacterial contaminants and collected by centrifugation for 15 min at 3000×g (Model 5810R, Eppendorf, Hamburg, Germany). The cells were resuspended in 1 mL TriReagent® and disrupted using a beadmill (FastPrep FP-120, Thermo Scientific, Karlsruhe, Germany) in presence of glassbeads (Ø∼200 µm). Phase separation took place after addition of chloroform; RNA, DNA and proteins were precipitated stepwise from aqueous and organic phase by addition of isopropanol, ethanol and acetone, respectively. Protein and RNA was washed according to manufacturer’s protocol. Protein pellets were resuspended in 20 mM Tris-HCl buffer, pH 7.4 containing 1% SDS. RNA was resuspended in RNAse-free water (Qiagen, Hilden, Germany). Subsequently, the protein content was estimated with the BCA (bichinoacid assay)- method (Pierce/Thermo Scientifc, Bonn, Germany) and the samples were stored at −20°C for later application. RNA purity, yield and integrity were assessed using Nanodrop ND-1000 (PEQLAB, Erlangen, Germany) and 2100 Bioanalyzer (Agilent, Stuttgart, Germany) and stored for later application at −80°C.

### Identification and Analysis of PKS Sequences

In order to identify novel PKS candidates in dinoflagellates, the protein sequence of the previously found *Karenia brevis* KS was blasted using tblastx implementation at the NCBI website against the total set of dinoflagellate ESTs in the NCBI sequence collection. A total number of three *A. ostenfeldii* KS (accession numbers: Ac0019: JX068836, Ac0038: JX068837, 10-x_j14: JX068838) and two *H. triquetra* KS ESTs (Accessionnumber: HTE5908: JX068839, HTE6310:JX068840) were collected and used to design gene specific Primers within the CLC main workbench (CLC Bio, Aarhus, Denmark) for rapid amplification of cDNA PCR to obtain full length transcript contigs.

cDNA synthesis was performed with 1 µL Superscript III reverse transcriptase (200 U/µL) and 500 ng total RNA from *A. ostenfeldii* AOSH 2 and *H. triquetra* K-0481 in presence of either 1µl random hexamer primers (50 ng/µL) or a 1 µL special adapter ligated oligo dT primer (50 µM) (GCTGTCAACGATACGCTACGTAACGGCATGACAGTGTTTTTTTTTTTTTTTTTTTTTTTT) according to the manufacturer’s recommendations (Invitrogen, Darmstadt, Germany). Random hexamer primers or the oligo dT primer were chosen when the later experiment was rapid amplification of 5′ or 3′ end, respectively.

For 3′ RACE PCR, gene specific primers (GSP) were designed according to the GeneRacer manual’s suggestions (23–28 nt, 50–70%GC, Tm ≥72°C) (Table S1.). The PCR was carried out in 50 µL reactions with adaptor primer directing the poly A tail adaptor, GSP, cDNA, nuclease free water, 2× Type-it multiplex PCR master mixture and Q-solution (Qiagen, Hilden, Germany). A gradient touchdown PCR approach was chosen and the cycling parameters were as follows: 94°C for 2 min and 5 cycles at 94°C for 30 s and 72°C±2°C for 3 min followed by 5 cycles at 94°C for 30 s and 70°C±2°C for 3 min and then 25 cycles at 94°C for 30 s, 68°C±2°C for 30 s and 72°C for 3 min. PCR products were separated on 1% agarose and the highest bands were excised under blue light and gel purified using the MiniElute Gel extraction kit (Qiagen, Hilden, Germany).

For 5′ RACE PCR, a different approach was chosen. The spliced leader sequence (CCGTAGCCATTTTGGCTCTTG, 21 nt, Tm = 64°C) was used as the forward primer and the respective reverse GSP has been designed accordingly (19–22 nt, Tm = 62−64°C) (Table S1). The PCR was carried out in 50 µL reaction with the 3 µL 10 µM spliced leader primer, 1µL 10 µM reverse GSP, 1 µL cDNA, 1 µL dNTP solution, 1 µL (5 U/µL) Platinium Hot Master Taq polymerase nuclease free water and 5 µL10×PCR buffer (5PRIME, Hamburg, Germany). A gradient touch down PCR approach was chosen and the cycling parameters were as follows: 94°C for 2 min and 5 cycles at 94°C for 30 s and 64°C±2°C and 68°C for 4 min followed by 5 cycles at 94°C for 30 s and 62°C±2°C and 68°C for 4 min and then 25 cycles at 94°C for 30 s and 60°C±2°C and 68°C for 4 min and a final extension for 10 min at 68°C. PCR products were separated on 1% agarose and the highest bands were excised under blue light and gel purified using the MiniElute Gel extraction kit (Qiagen, Hilden, Germany).

All RACE products were cloned using the TOPO TA cloning kit (Invitrogen, Darmstadt,Germany) and transformed into One Shot® TOP10 electrocompetent *E. coli* cells (Invitrogen, Darmstadt Germany) following the manufacturer’s protocols. The obtained clones were checked for the expected insert in a 50 µL PCR reaction with 1 µL cell lysate, 1 µL M13 F primer, 1 µL 10 µM M13 R, 1 µL cDNA, 1 µL dNTP solution, 1 µL (5 U/µL) Platinium Hot Master Taq polymerase nuclease free water and 5 µL10×PCR buffer (5PRIME, Hamburg, Germany) with the following cycling parameters: 94°C for 2 min and 25 cycles with 94°C for 1 min, 68°C for 1 min and 55°C for 3 min and a final round of extension at 68°C for 10 min. The PKS candidates were then sequenced on a 3130*xl* genetic analyzer (Applied Biosystems, Darmstadt, Germany) according to the manufacturer’s instructions. Internal sequencing primers were designed in order to close the contigs (Table S1) and applied using the same protocols described above.

The obtained nucleotide sequences were aligned with the referring PKS-EST to construct full length transcript contigs by using the CLC main workbench (CLC Bio, Aarhus, Denmark). *In silico* translation was done with the open reading frame finder plug-in and the virtual ribosome implemented into the program. Amino acid sequences were then annotated using the PFAM domain search [28] and NCBI’s BLASTx function [29].

### Phylogenetic Analysis

Amino acid sequences of KS domains were obtained from NCBI Genbank and John et al (2008) [10]. In total, 59 Type I and Type II KS sequences were used in a maximum likelihood phylogenetic analysis. These represent the major clades from prokaryotes, fungi, animals, apicomplexa, haptophytes and chlorophyte PKS evolution. This data set was augmented by the translated KS sequences from *K. brevis* and the dinoflagellate KS sequences described here. Multiple amino acid alignment was performed with the MUSCLE algorithm [30] included in MEGA 5 [31]. A maximum likelihood phylogenetic tree was generated with PhyML 3.0 [32] using a Le-Gascuel amino acid replacement matrix [33] and likelihood ratio test [34] and 100 bootstrap analyses were performed as a measure of credibility for each branch. Subsequently, the output tree was processed and edited with MEGA 5.0. The multiple alignment is provided in the supplementary data section (Fig. S3).

### Analysis of the N- and C-terminal Region

In order to identify the origin of the N- and C- terminal regions of dinoflagellate KS sequences, the respective regions were truncated from the adjacent KS domain and blasted against the NCBI and PFAM database. No significant hit beside the known *K. brevis* KS was obtained after BLASTx analysis. Multiple alignment and phylogenies of the truncated sequences were calculated as described above for the KS domains.

### Immunoblotting

10 µg total protein was separated on 10% SDS/polyacrylamide gel and transferred onto polyvinylidene fluoride membranes by Western Blot. Membranes were blocked with 5% skimmed milk in Tris buffered saline with 0.5% Tween 20 (TBS-T) for 1 h. Primary antibodies (Rabbit anti-*Karenia brevis* KS KB 2006 1:5000) were diluted in 5% skimmed milk in TBS-T and the membranes were incubated at 4°C overnight. Subsequently, the membranes were washed three times with TBS-T and incubated with the appropriate secondary antibody dilution (Goat anti-*rabbit* 1∶20000 Peroxidase-conjugated) (Sigma-Aldrich, Schnelldorf, Germany) in 5% skimmed milk/TBS-T for 2 h at room temperature.

Western Blots: Custom peptide polyclonal antibodies specific for *Karenia brevis* KS domain (KB2006; GenBank accession no. EF410007) were kindly provided by Dr. Frances Van Dolah (NOAA Center for Coastal and Environmental Health and biomolecular research, Charleston, South Caroline, USA).

Sequence accession numbers: The PKS transcripts identified in this manuscript have been submitted to GenBank under the following accession numbers: Ac0019 JX068836, Ac0038 JX068837, 10-x_J14 JX068838, HTE5908 JX068839 and HTE6310 JX068840.

## Supporting Information

Figure S1 **Presence of KS proteins in A. ostenfeldii and Heterocapsa triquetra**. Western blot analysis of protein extracts from *A. ostenfeldii* AOSH 2 and *Heterocapsa triquetra* SCCAP strain K-0481 using a polyclonal rabbit anti- *K. brevis* KS antibody. Note that the size of the labelled polypeptide is consistent with the molecular weight predicted based on the transcript sequences, confirming expression as monofunctional units.
(TIF)

Figure S2 **Extended phylogenetic tree of Type I and Type II KS domains from prokaryotic and eukaryotic PKS and FAS**. Sixty-eight taxa representing Type I and Type II KS domains were retrieved from NCBI genbank, John et al., 2008 and Lopèz-Legentil et al., 2010 and analyzed by a maximum likelihood approach. Multiple amino acid alignment was performed with the MUSCLE algorithm included in MEGA 5.0. Subsequently, a maximum likelihood phylogenetic tree was generated and processed with MEGA 5.0 using a Jones-Taylor-Thornton amino acid substitution matrix and 100 bootstrap analyses were performed as a measure of credibility for each branch. The Type II KS and the acyl carrier protein synthases (ACPS) were used as outgroup. Bootstrap values ≥50% are marked on appropriate branches. Overall tree topology is in accord with previous reports [5], [10]. Dinoflagellate KS ( [11]; This report) is classified as a well supported group within the protistan Type I FAS/PKS clade. Additional sequences from different cyanobacteria and *Karenia brevis* sequences which were added upon the reviewer’s request cluster mainly with modular Type I PKS trans AT but neither with KS reported here nor with Monroe & van Dolah *K. brevis* KS sequences.
(TIFF)

Figure S3 **Multiple alignment of Type I and Type II KS domains from prokaryotic and eukaryotic PKS and FAS.** Fifty-nine taxa representing Type I and Type II KS domains were *in silico* translated and aligned using the MUSCLE algoritm included into MEGA 5.0. If no GenBank accession numbers provided information can be get from *John U, Beszteri B, Derelle E, Van de Peer Y, Read B, et al. (2008) Novel insights into evolution of protistan polyketide synthases through phylogenomic analysis. Protist 159: 21–30. doi:10.1016/j.protis.2007.08.001.
(TXT)

Table S1 **Primer collection used to obtain the 5 full length KS transcripts.** Method abbreviations: S –Sanger Sequencing; 5′RACE-Rapid amplification of 5′ cDNA ends; 3′RACE-Rapid amplification of 3′ cDNA ends.
(DOCX)
