# Supplementary figures and images for: Putative Monofunctional Type I Polyketide Synthase Units: A Dinoflagellate-Specific Feature?
Source: PLoS One. 2012 Nov 5;7(11):e48624. doi: 10.1371/journal.pone.0048624 (PMC3489724; doi:10.1371/journal.pone.0048624)

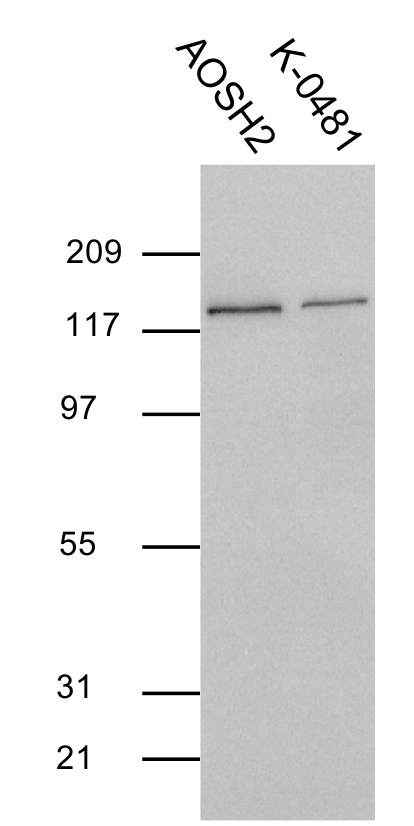

Supplement: Figure S1 — Presence of KS proteins in A. ostenfeldii and Heterocapsa triquetra. Western blot analysis of protein extracts from A. ostenfeldii AOSH 2 and Heterocapsa triquetra SCCAP strain K-0481 using a polyclonal rabbit anti- K. brevis KS antibody. Note that the size of the labelled polypeptide is consistent with the molecular weight predicted based on the transcript sequences, confirming expression as monofunctional units. (TIF) [file pone.0048624.s001.tif]

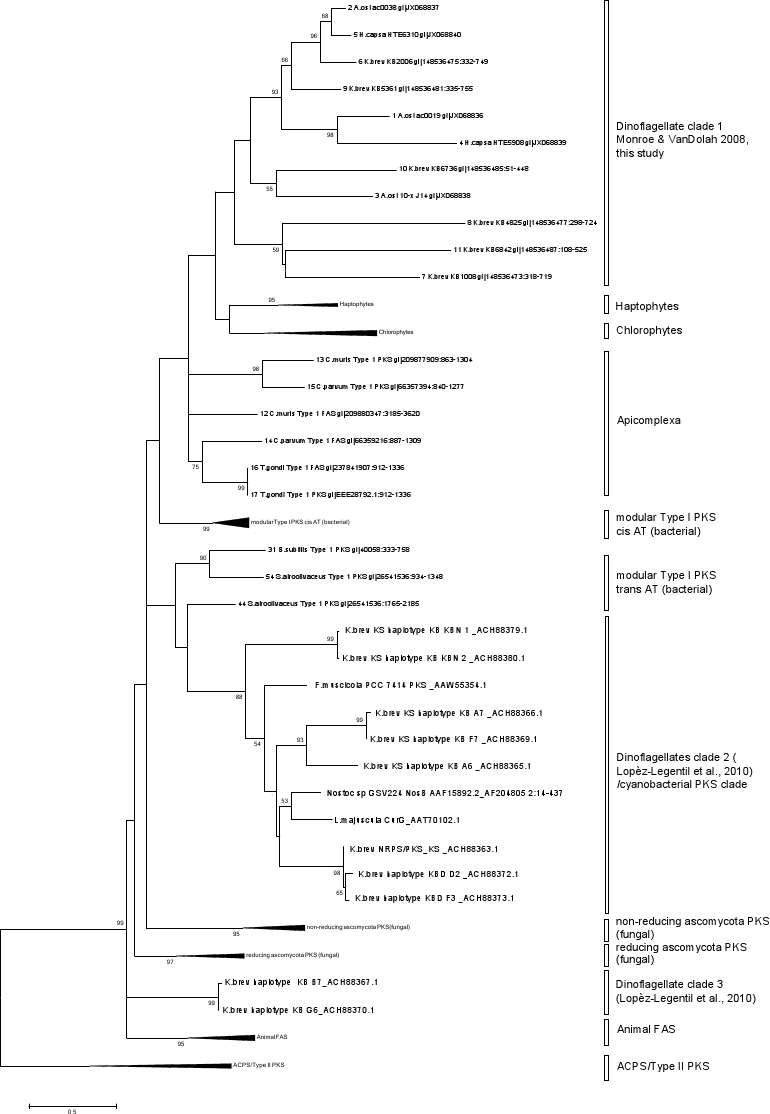

Supplement: Figure S2 — Extended phylogenetic tree of Type I and Type II KS domains from prokaryotic and eukaryotic PKS and FAS. Sixty-eight taxa representing Type I and Type II KS domains were retrieved from NCBI genbank, John et al., 2008 and Lopèz-Legentil et al., 2010 and analyzed by a maximum likelihood approach. Multiple amino acid alignment was performed with the MUSCLE algorithm included in MEGA 5.0. Subsequently, a maximum likelihood phylogenetic tree was generated and processed with MEGA 5.0 using a Jones-Taylor-Thornton amino acid substitution matrix and 100 bootstrap analyses were performed as a measure of credibility for each branch. The Type II KS and the acyl carrier protein synthases (ACPS) were used as outgroup. Bootstrap values ≥50% are marked on appropriate branches. Overall tree topology is in accord with previous reports [5], [10]. Dinoflagellate KS ( [11]; This report) is classified as a well supported group within the protistan Type I FAS/PKS clade. Additional sequences from different cyanobacteria and Karenia brevis sequences which were added upon the reviewer’s request cluster mainly with modular Type I PKS trans AT but neither with KS reported here nor with Monroe & van Dolah K. brevis KS sequences. (TIFF) [file pone.0048624.s002.tiff]
