# Supplementary material for: Putative Monofunctional Type I Polyketide Synthase Units: A Dinoflagellate-Specific Feature?
Source: PLoS One. 2012 Nov 5;7(11):e48624. doi: 10.1371/journal.pone.0048624 (PMC3489724; doi:10.1371/journal.pone.0048624)
Supplement: Table S1 — Primer collection used to obtain the 5 full length KS transcripts. Method abbreviations: S –Sanger Sequencing; 5′RACE-Rapid amplification of 5′ cDNA ends; 3′RACE-Rapid amplification of 3′ cDNA ends. (DOCX) [file pone.0048624.s004.docx]

Table S1: Primer collection used to obtain the 5 full length KS transcripts. Method abbreviations: S –Sanger Sequencing ; 5’RACE-Rapid amplification of 5’ cDNA ends; 3’RACE-Rapid amplification of 3’ cDNA ends.

| Primer | Sequence | Method |
| --- | --- | --- |
| Ac0038_S_1 | CCAGCCGCTATGAGTACA | S |
| Ac0019_S_1 | TTCCGCATCATCCACTTCA | S |
| Ac0019_S_2 | GATCTCAAGAGGCAGCAG | S |
| Ac0038_S_2 | CATCCAGTCGTCCTTGTC | S |
| Ac0019_S_3 | TCCAACGCAATTCTCACAG | S |
| Ac0019_S_4 | GTCGTGATTGGTGATCTTC | S |
| HTE6310_S_1 | AACACTTGAAGAAGCCAC | S |
| HTE6310_S_2 | GCTGGGAAGGCACAATCA | S |
| 10-x_J14_S_1 | TACGTCAAGGGCATCTC | S |
| 10-x_J14_S_2 | GTGCGCGGTCATTGAGA | S |
| HTE5908_S_1 | TGAAGAACGGCGAGGAG | S |
| HTE5908_S_2 | CCGAACGAGGAAATGTGGA | S |
| HTE5908_S_3 | TCATCTTCAACCTCAAAGGC | S |
| Ac0038_rev | TGAACAAGCCGCAGAAACCAG | 5'RACE |
| Ac0019_rev | ACGAGAGACGAAGAGCAAGCA | 5'RACE |
| 10-X_J14_rev | GGAACCACCAAAGCCGAAGG | 5'RACE |
| HTE6310_rev | TGCGGCTGTTGACCATGATGA | 5'RACE |
| HTE5908_rev | CGTTGGCAGAATCGTTGAAGG | 5'RACE |
| 10-X_J14_for | GTGTCTTCCTTCGGCTTTGGTGGT | 3'RACE |
| Ac0019_for | AACGGACCCGCCCAGAGAGAAGT | 3'RACE |
| Ac0038_for | TGCCAGAGTACGGCGCATGAGTTC | 3'RACE |
| HTE6310_for | GTGCTTACACCAAGCACGGCTCCTT | 3'RACE |
| HTE5908_for | ACGAACAAGACCACAACCGGCCAC | 3'RACE |
